# Tick-Virus Interactions: Toll Sensing

**DOI:** 10.3389/fcimb.2017.00293

**Published:** 2017-06-30

**Authors:** Nicholas Johnson

**Affiliations:** ^1^Animal and Plant Health AgencyAddlestone, United Kingdom; ^2^Faculty of Health and Medicine, University of SurreyGuildford, United Kingdom

**Keywords:** ticks, Toll, Toll-like receptors, virus, immunity

## Abstract

Ticks are important vectors of viruses that infect and cause disease in man, livestock, and companion animals. The major focus of investigation of tick-borne viruses has been the interaction with the mammalian host, particularly the mechanisms underlying disease and the development of vaccines to prevent infection. Only recently has research begun to investigate the interaction of the virus with the tick host. This is striking when considering that the virus spends far more time infecting the tick vector relative to the vertebrate host. The assumption has been that the tick host and virus have evolved to reach an equilibrium whereby virus infection does not impede the tick life cycle and conversely, the tick does not restrict virus replication and through blood-feeding on vertebrates, disseminates the virus. The development and application of new technologies to tick-pathogen interactions has been fuelled by a number of developments in recent years. This includes the release of the first draft of a tick genome, that of *Ixodes scapularis*, and the availability of tick-cell lines as convenient models to investigate interactions. One of the by-products of these investigations has been the observation of familiar proteins in new situations. One such protein family is Toll and Toll-like receptors that in vertebrates play a key role in detection of microorganisms, including viruses. But does Toll signaling play a similar role in detection of virus infection in ticks, and if it does, how does this affect the maintenance of viruses within the tick?

## Tick-microorganism coexistence

The phylum Arthopoda emerged during the “Cambrian explosion” (540–485 million years ago) creating numerous groups, many that have survived to the present day. One of these, the Chelicerata, contains the order Acari, which in turn contains species that obtain nutrition through blood feeding on vertebrates, collectively termed ticks. Fossil records indicate that ticks have been present from at least the Cretaceous period (146–65 million years ago) where they could feed on mammals (de la Fuente, 2003), and likely evolved earlier to take blood meals from reptiles and then birds (Nava et al., 2009). Irrespective of the precise date that hematophagous behavior evolved, it is clearly measured in millions of years and implies a long period over which ticks were in turn parasitized by microorganisms (viruses, bacteria, and protozoa) that are found in abundance in ticks extant today (Vayssier-Taussat et al., 2015). The presence of microorganisms in ticks appears to have little impact on the tick, although presumably there is an energetic cost to harboring such microorganisms. Some authors have characterized this as a combination of conflict and cooperation (de la Fuente et al., 2016). However, recent studies demonstrated that ticks do respond in a coordinated fashion to infection with pathogens of mammals (Alberdi et al., 2016), at least in order to control infection if not eliminate it.

One of the major groups of microorganisms associated with transmission by ticks is the viruses (Labuda and Nuttall, 2004). The interaction of most tick-borne viruses with vertebrate hosts leads to a transient infection that causes morbidity and mortality. Occasionally, viruses are found that appear avirulent in humans, such as the flavivirus Langat virus (LGTV), although these are the exception and provide a useful model for more virulent viruses (Tsetsarkin et al., 2016). Infection with a virulent virus in a vertebrate host is usually short-lived and, if the host survives, eliminated by the rapid induction of antibodies and subsequent development of cell-mediated responses. By contrast, the interaction with the tick appears more benign and long-lived (Nuttall, 2009). Indeed, for ticks to act as the reservoir for viruses, the virus must persist in the tick for long periods, potentially years, without harming the tick or preventing completion of its various life stages. In addition, viruses can be transmitted transovarially to the next generation of ticks.

Such a harmonious arrangement contrasts completely with the virus-vertebrate interaction and recent investigations suggest that the virus-tick relationship is more dynamic. Preliminary findings have demonstrated both transcriptomic and proteomic responses to infection with flaviviruses such as tick-borne encephalitis virus (TBEV) and LGTV (Weisheit et al., 2015). A subsequent proteomics study of *Ixodes scapularis* cells infected with LGTV demonstrated increased expression of proteins associated with metabolic pathways (Grabowski et al., 2016). This may represent a cellular response to stress or manipulation, by the virus, of the host cells metabolic machinery. What other potential responses does the tick have in response to virus infection?

## Antiviral responses in ticks

Arthropods have an array of antiviral mechanisms to prevent and control infection (reviewed by Kopáček et al., 2010). These include RNA interference (Schnettler et al., 2014), antiviral peptides such as defensins (Talactac et al., 2017) and detection through Toll receptors (Rükert et al., 2014). This last group have been extensively studied in vertebrates. Toll-like receptors (TLRs) are a recognized family of pattern-recognition receptors that form part of the innate immune system of vertebrates (Akira and Takeda, 2004). In addition to binding to a diverse range of pathogen motifs, they also provide a signaling function that activates immune responses to infection. A distinctive feature of the TLRs is their conserved structure composed of an N-terminal leucine-rich repeat (LRR) ectodomain, a transmembrane domain, and toll-interleukin receptor (TIR) signaling domain (Bell et al., 2003). Multiple LRRs, ranging from 19 to 25 in human TLRs, create a long stretch of beta-sheet that forms a horseshoe-shaped structure that enables pattern-recognition (Botos et al., 2011). The importance of TLRs to the control of infection is highlighted by the widespread presence of these proteins in both invertebrates and vertebrates (Buchmann, 2014).

Toll-like proteins evolved early in the evolution of life and the proteins present in extant species can be found in most multicellular organisms, including many ancient invertebrates (Buchmann, 2014). This is not the case for all innate immune proteins. RIG-like receptors (RLRs), including proteins such as RIG-I, LGP2, and MDA5, have not been found in arthropod genomes although they are present in other invertebrate animals, suggesting the early loss of RLR precursors in the phylum's evolution (Mukherjee et al., 2013). However, TLR genes are often present in numerous copies within the genome of many species and have evolved to fulfill a number of roles including structural development (Anderson et al., 1985) and immunity against pathogens, including viruses (Ferreira et al., 2014). However, the mechanism of action of arthropod Toll differs from the pattern recognition receptor function of mammalian TLRs. Insect Toll is activated as a result of cleavage of an endogenous ligand protein, Spätzle, following engagement with carbohydrates of microbial origin (Arnot et al., 2010). Cleavage causes conformation change in Spätzle enabling it to engage with the Toll receptor. It is likely that Toll functions through a similar mechanism in ticks but what is the evidence for this?

## A role for toll in tick antiviral responses

Firstly, is there a gene encoding tick Toll in the tick genome? The completion of a detailed draft of the *I. scapularis* genome (Gulia-Nuss et al., 2016) suggests that multiple isoforms of tick Toll exist. A recent review on the subject of immunity genes in *I. scapularis* reported 13 copies of *Toll* genes and 2 copies of the *Spätzle* gene (Smith and Pal, 2014). This compares favorably with the nine Toll receptors that exist in *Drosophila melanogaster* (Arnot et al., 2010). However, it is too early to assume that all of the genes identified in ticks produce a functional protein and have a role in immunity. Some may be pseudogenes or produce proteins with a developmental function.

**Figure 1:**
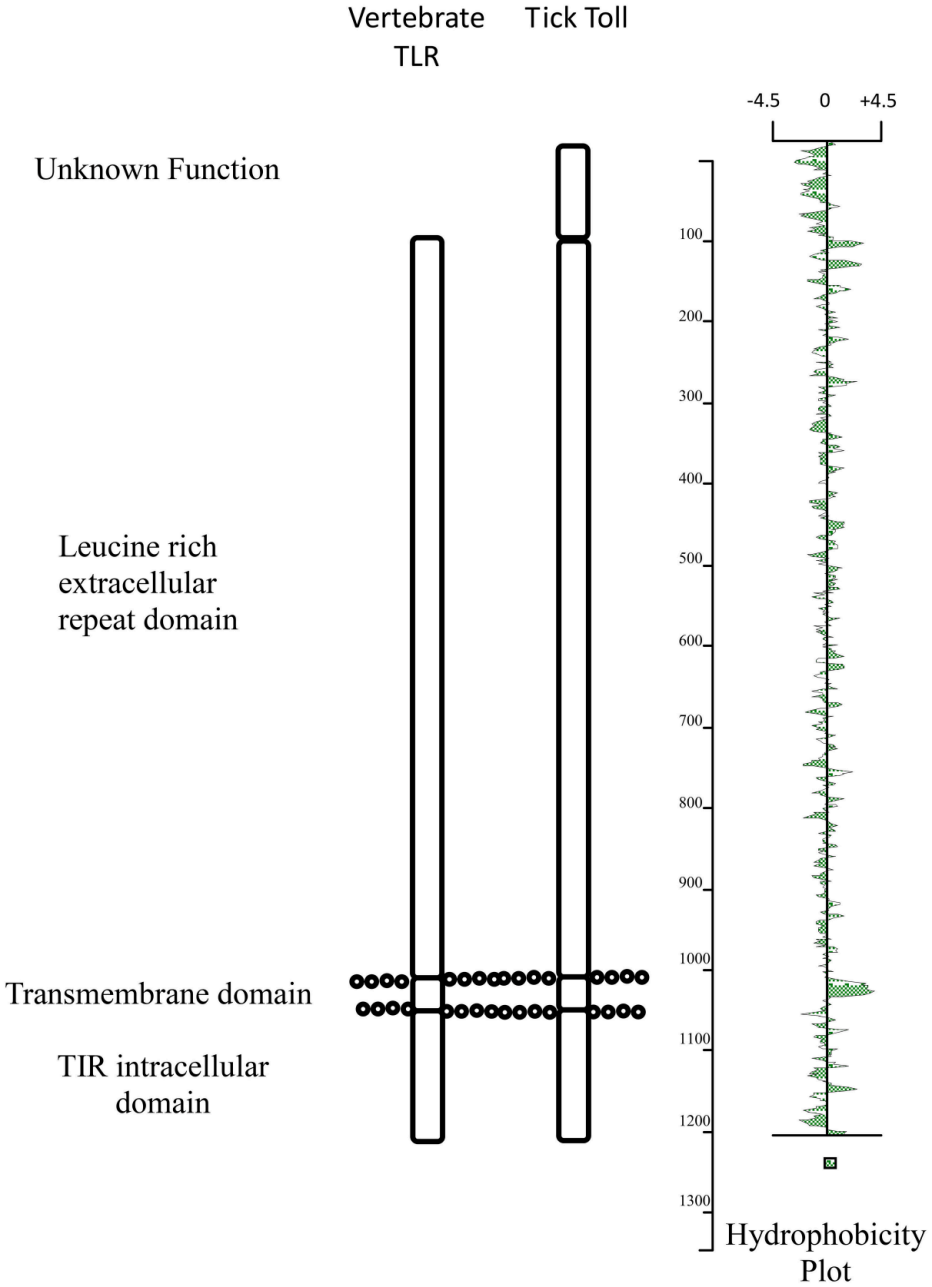
Schematic comparison of the main structural domains present in *Ixodes ricinus* toll (ISWC02240) and human TLR3.

An alternative approach is to measure Toll activity in ticks in response to microbial infection. Mansfield et al. (2017) have recently compared the transcriptional response to infection of *Ixodes ricinus* cells with a bacterium, *Anaplasma phagocytophilum*, to infection with two flaviviruses, louping ill virus (LIV) and TBEV. One striking observation from this study was the up-regulation of a single Toll-like protein transcript (ISCW022740) following infection with the viruses but not the bacterium. Three other Toll transcripts showed very little change to infection with the pathogens. This suggests that transcript ISCW022740 may play a role in the antiviral response. This strongly mirrors the mammalian response to infection where Toll-like receptors such as TLR3 are upregulated in response to infection (McKimmie et al., 2005). Structurally, the protein encoded by transcript ISCW022740 shares many of the characteristics of TLRs including a large leucine rich domain composed of numerous LRR motifs, a transmembrane domain and a putative TIR domain (Figure 1). In addition, there appears to be a one hundred amino acid domain at the amino terminus of the protein (extracellular) that increases the size of tick toll in comparison with mammalian TLRs. The role of this extra domain is unknown although appears to be shared with other arachnid toll proteins and may play a role in engaging with the homolog of the Spätzle protein.

## Conclusions

The association between ticks and viruses is a fascinating one and a growing field of investigation. Ticks harbor a vast array of endogenous viruses (Bell-Sakyi and Attoui, 2013; Li et al., 2015). However, it is not clear what impact this infection has on the tick and there is little evidence that this impact is deleterious in the way that certain viruses are to insects (Carlson et al., 2006; Chen and Siede, 2007; Xu and Cherry, 2014). Ticks encode Toll proteins and there is early evidence that at least one of these proteins could play some role in the tick response to virus infection. This may take the role of actively controlling virus and *in vitro* infection of tick cells with tick-borne viruses shows no apparent cellular changes in stark contrast to the lytic cytopathic effect observed in many mammalian cells infected with the same virus. However, an alternative interpretation could be that infection stresses the cell. Cell-lines used in such studies are often derived from embryonic tissue and stress could lead to induction of transcripts associated with a developmental response. Ticks appear to tolerate virus infection but further investigation is required to understand what mechanisms tick cells use to control virus infection and why this does not lead to elimination of the virus analogous to the response in vertebrate hosts.

## Author contributions

The author confirms being the sole contributor of this work and approved it for publication.

### Conflict of interest statement

The author declares that the research was conducted in the absence of any commercial or financial relationships that could be construed as a potential conflict of interest.
